# Can Randall’s plug composed of calcium oxalate form via the free particle mechanism?

**DOI:** 10.1186/s12894-017-0274-7

**Published:** 2017-09-08

**Authors:** F. Grases, O. Söhnel

**Affiliations:** 1 Laboratory of Renal Lithiasis Research, University Institute of Health Sciences Research(IUNICS), University of Balearic Islands, Palma of Mallorca, Spain; 2 University of J.E. Purkyně, Faculty of Environmental Studies, Ústí n.L, Czech Republic

**Keywords:** Randall’s Plug, Calcium oxalate monohydrate, Formation mechanism

## Abstract

**Background:**

The likelihood of a Randall’s plug composed of calcium oxalate monohydrate (COM) forming by the free particle mechanism in a model of kidney with a structure recently described by Robertson was examined at the most favourable conditions for the considered mechanism.

**Methods:**

The Robertson model of the kidney is used in the following development. The classical theory of crystallization was used for calculations.

**Results:**

Initial COM nuclei were assumed to form at the beginning of the ascending loop of Henle where the supersaturation with respect to COM has been shown to reach the threshold level for spontaneous nucleation. Nucleation proceeds by a heterogeneous mechanism. The formed particles are transported in the nephron by a laminar flow of liquid with a parabolic velocity profile. Particles travel with a velocity dependent on their position in the cross-section of the nephron assumed to be straight tubule with smooth walls and without any sharp bends and kinks. These particles move faster with time as they grow as a result of being surrounded by the supersaturated liquid. Individual COM particles (crystals) can reach maximum diameter of 5.2 × 10^−6^ m, i.e. 5.2 μm, at the opening of the CD and would thus always be washed out of the CD into the calyx regardless of the orientation of the CD. Agglomeration of COM crystals forms a fractal object with an apparent density lower than the density of solid COM. The agglomerate that can block the beginning of the CD is composed of more crystals than are available even during crystaluria. Moreover the settling velocity of agglomerate blocking the opening of the CD is lower than the liquid flow and thus such agglomerate would be washed out even from upward-draining CD.

**Conclusions:**

The free particle mechanism may be responsible for the formation of a Randall’s plug composed by COM only in specific infrequent cases such as an abnormal structure of kidney. Majority of incidences of Randall’s plug development by COM are caused by mechanism different from the free particle mechanism.

**Electronic supplementary material:**

The online version of this article (doi: 10.1186/s12894-017-0274-7) contains supplementary material, which is available to authorized users.

## Background

Renal papillary calcifications can be classified into two distinct groups [1]. One type results from a subepithelial calcification (hydroxyapatite) of the renal papillary tissue as a consequence of a previous injury. The disruption of the papillary epithelial layer by biological hydroxyapatite plaque becomes the nidus of a calcium oxalate monohydrate (COM) papillary calculus. The other type of papillary calcification results from occlusions of the openings of the ducts of Bellini by stone-forming crystals (so-called Randall’s plugs). Randall’s plugs are usually composed of calcium oxalate monohydrate and/or dihydrate (COD), biological hydroxyapatite and organic matter, and occur in the terminal collecting ducts. These Randall’s plugs can be also related to COD calculi or COM papillary calculi formed on papillary tips.

Two mechanisms for blocking collecting ducts by solid concretions, i.e. Randall’s plug, have been suggested, the free and fixed particle mechanisms [2]. The free particle mechanism assumes that during the residence time of urine in the nephron and collecting duct a spherical object that can obstruct the opening into the calyx develops as a result of growth of a single particle or by agglomeration of smaller particles. The fixed particle mechanism assumes that a large concretion develops on a solid nidus firmly attached to the collecting duct wall. A crystal of calcium oxalate (CaOx) formed in urine during its residence time in the nephron attached to a Bellini’s duct or to subepithelial calcification (hydroxyapatite) of the renal papillary tissue followed by an injury can serve as such a solid nidus.

Formation of a Randall’s plug by the free particle mechanism was generally considered to be highly improbable. Recently, Robertson implemented previously omitted hydrodynamic factors into the original free particle model and concluded that this mechanism can play a significant role in the formation of a Randall’s plug [3, 4]. The additional hydrodynamic factors implemented were (i) the spatial velocity profile of a liquid flowing through a tube, (ii) the residence time of a particle in a tube as a function of its position, (iii) the drag effect of the tubular walls on the particle and (iv) the effect of gravity on crystals in upward-draining tubules. Implementation of these additional factors into the model of the free particle mechanism and the resulting conclusions were, however, only qualitatively described in the paper. The aim of the current work was to rigorously determine the feasibility of the free particle mechanism under the most favourable conditions for formation of a Randall’s plug composed of COM when taking into consideration the hydrodynamic factors recently introduced by Robertson.

## Methods

### Kidney

Robertson model of the kidney [3] is used in the following calculations. This model describes the ascending Henle loop as a circular truncated cone with average length L_HL_ of 1.2 × 10^−2^ m and internal radius r_LH_ increasing from 1.12 × 10^−5^ to 1.20 × 10^−5^ m, the following distal tubule as a cone with a length L_D_ of 6 × 10^−3^ m and internal radius r_DL_ decreasing from 1.2 × 10^−5^ to 1 × 10^−5^ m, and the collecting duct (CD) as a cone with a length L_CD_ of 2.7 × 10^−2^ m and internal radius r_CD_ increasing from 1 × 10^−5^ to 4 × 10^−5^ m at its opening at the papilla. The number of CDs per kidney is 320 and the number of the nephrons per kidney is 1.31 × 10^6^. Thus 4094 nephrons are attached to each CD in this model.

### Urine

Liquid passing the ascending Henle loop and distal tubule is approximately ten times more diluted than the final urine leaving the collecting duct [3]; that is the volume of liquid passing though the Henle loop and distal tubule is ten times the volume of urine produced. A volume of 1.5 × 10^−3^ m^3^ is considered to be an average diuresis per day of adults. The volume of urine produced between the morning and evening is two to four times that at night. The minimum night production of urine is thus 0.30 × 10^−3^ m^3^ per 8 h by two kidneys [5, 6] and this value was used in calculations.

The density and dynamic viscosity of the liquid (i.e. ten-fold diluted urine) in the nephron used for calculations were taken as values for water at 37 °C, i.e. the density ρ = 994 kg m^−3^ and the dynamic viscosity μ = 6.91 × 10^−4^ kg m^−1^ s^−1^. The density of urine in the CD is 1015 kg m^−3^ at 37 °C [7] and the kinematic viscosity at 37 °C is ν = 0.829 × 10^−6^ m^2^ s^−1^ [8]. The dynamic viscosity of urine is μ = ν x ρ = 8.41 × 10^−4^ kg m^−1^ s^−1^.

### Characteristics of liquid flow in tubules

The liquid flow in tubules at night was calculated from data in the Kidney section using expressions in Additional file 1, see Table 1.

**Table 1:** Characteristics of liquid flow in tubules at night

		Ascending Henle loop	Distal tubule	Collecting duct
Volume	V (m^3^)	5.07 × 10^−12^	2.29 × 10^−12^	5.94 × 10^−11^
Average radius	r (m)	1.16 × 10^−5^	1.10 × 10^−5^	2.65 × 10^−4^
Volumetric flow	Q (m^3^ s^−1^)	3.98 × 10^−14^	3.98 × 10^−14^	1.63 × 10^–11#^
Average linear velocity	u_av_ (m s^−1^)	9.42 × 10^−5^	1.05 × 10^−4^	3.24 × 10^–3#^
Average transit time of liquid	t_tr_ (s)	127	57	0.8 (3.6^*^)
Reynolds number	Re (−)	3.2 × 10^−3^	3.3 × 10^−3^	2.1 (0.31﻿^#﻿^)

Value denoted by asterisk is the transit time if volumetric flow in the CD is equal to the flow rate at the opening, ^#^ value at the opening of the CD

The flow of liquid is purely laminar in the ascending Henle loop and distal tubule as indicated by the Reynolds number Re « 1 and laminar in the CD where the average Reynolds number is Re ~ 2. The parabolic velocity profile of liquid in laminar flow is described by

1 $$ \mathrm{u}\left(\mathrm{R}\right)=2\ {\mathrm{u}}_{\mathrm{av}}\left[1\hbox{--} {\left(\mathrm{R}/\mathrm{r}\right)}^2\right] $$

where r is the radius of the tubule and R is the distance from the tubule axis.

**Fig. 1:**
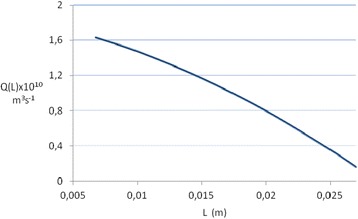
The volumetric liquid flow through the CD as a function of distance, L, from its beginning

The hydrodynamics in the CD is rather complicated. Nearly all nephrons are connected to the CD at its upper section [9]. Assuming that all nephrons are connected to the CD in the first quarter of its length, then the area available for water absorption would be the inner surface of three quarters of the CD length. The finding of Robertson that concentration of solute in the liquid entering the CD is ten times less than that in the final urine [3] means that 90% of the liquid entering the CD must be reabsorbed in the CD and hence the volumetric liquid flow decreases along the CD, see Fig. 1.

The average volumetric flow rate is Ave[Q(L)] = 7.58 × 10^−11^ m^3^ s^−1^, the average linear velocity of liquid is 3.45 × 10^−2^ m s^−1^ and the transit time of liquid through CD is 0.8 s (Additional file 2).

### Supersaturation

Supersaturation S of a liquid in the nephron with respect to COM that was used for the current study was derived from Fig. 1 in [4] using the highest reported level of plasma oxalate concentration 2.75 × 10^−6^ mol L^−1^. Supersaturation in the ascending loop of Henle has been shown to linearly decrease from 14 to 7, to be 7 in the distal tubule and to parabolically increase from 7 to 270 in the CD.

### Nucleation

The onset of spontaneous nucleation of COM in an aqueous solution was shown to occur at a supersaturation 9 [10], 10 [11] and 14 [3]. Formation of primary particles of CaOx (by heterogeneous nucleation on present organic debris) with an initial diameter of 0.1 × 10^−6^ m [3, 12] was assumed to take place at the beginning of the ascending Henle loop (in accordance with Robertson model).

### Crystal growth

The rate of crystal growth is usually expressed as the increase of radius or diameter D of a spherical particle with volume equal to volume of an average particle per unit of time. The growth rate equation of COM crystal was calculated using the equation [13]

2 $$ \mathrm{dD}/\mathrm{dt}=1.12\mathrm{x}{10}^{-10}{\left(\mathrm{S}\hbox{--} 1\right)}^2\mathrm{m}\ {\mathrm{s}}^{-1} $$

## Results

### Transit time and size of COM crystals

COM primary particles formed at the beginning of Henle loop are assumed to be uniformly distributed throughout the volume of the liquid. For simplicity let consider the flow to be like a set of thin concentric shells of fluid sliding over one another. The liquid flow in the tubule is laminar with parabolic velocity distribution, see eq.(1); the velocity of the fluid at the centre line being twice the average velocity of the fluid and the velocity being zero at the first shell adjoining the tubular wall. The thickness of shell was assumed to be the same as the size of primary particles - 0.1 × 10^−6^ m. Particles at different position in the cross-section of the tubule therefore move with different velocities and thus have different transit times through the tubule. Because of these different transit times crystals attain different sizes at the end of the tubule.

Liquid surrounding CaOx particles is supersaturated with respect to COM and hence particles increase in size by regular crystal growth. Particle in the first stagnant shell adjacent to the tubular wall does not move but grows. When increment of particle size is 0.1 × 10^−6^ m, particle extends to the adjoining shell and then moves with the velocity of the liquid in this shell. Particle travels certain distance until its size due to growth reaches into the next shell and starts moving with velocity of this shell. The transit time through the tubules of a particle initially situated near the wall was calculated using eq.(III.5) in Additional file 3.

Along the ascending loop of Henle supersaturation linearly decreases from 14 to 7, as shown Fig. 1 of [4]. The average value of the function (S – 1)^2^ was calculated to be 94.33 (Additional file 1). The transit time of the slowest travelling spherical particle was calculated to be 358 s and during this time the particle was calculated to attain a diameter of 3.8 × 10^−6^ m. The transit time of a particle situated in the centre of the ascending Henle loop was calculated to be 64 s and during this time its diameter was calculated to increase to 0.7 × 10^−6^ m.

In the distal tubule supersaturation is 7 and thus a value of the function (S – 1)^2^ is 36. The transit time of the slowest moving particle entering from the ascending loop of Henle to the distal tubule was calculated to be 72 s and during this time the diameter of the spherical particle increased to 4.1 × 10^−6^ m. The transit time of a particle situated in the centre of the distal tubule was calculated to be 28 s. The diameter of a spherical particle entering the distal tubule from the centre of the ascending loop of Henle and travelling in its centre was calculated to increase at its end to 1.0 × 10^−6^ m.

When supersaturation with respect to COM at the CD exceeds approximately 14, particles of solid phase are formed by spontaneous nucleation followed by their fast growth. As a result supersaturation rapidly falls close to the threshold value. Let assume that supersaturation along the CD is 15 and the transit time of liquid through the CD 49 s quoted in [4] is equal to the transit time of crystals though, in fact this transit time was calculated to be much shorter (0.8 s). The diameter of the slowest moving spherical particle entering the CD from the distal tubule was calculated to increase to 5.2 × 10^−6^ m while the diameter of the fastest moving spherical particle (situated in the tubule axis) would increase to 2.1 × 10^−6^ m.

Minimum and maximum transit times, through a nephron and CD, of a crystal nucleated at the beginning of the ascending loop of Henle were calculated to be 141 s (2.3 min) and 479 s (8 min), respectively. The maximum diameter of an individual COM crystal appearing in the CD opening was calculated to be 5.2 × 10^−6^ m. Hence the collecting duct cannot be blocked by an individual COM crystal formed in the nephron.

### Agglomeration

Agglomerates are irregular spherical fractal objects as demonstrated in [14]. The apparent density of an irregular fractal agglomerate defined as the ratio of the mass of the agglomerate to the volume of a circumscribed sphere, i.e. occupied by agglomerate, decreases with its radius according to ρ_a'_~r_a_^Dm^ where D_m_ represents the mass agglomerate fractal dimension [15]. The more D_m_ deviates from 3 (Euclidean objects), the higher the degree of object fractality, i.e. the object becomes less regular. The value of D_m_ for a three dimensional fractal agglomerate formed by the random motion of particles that irreversibly associate with a growing germ has been shown to be 2.5 [16]. The ratio of the apparent density of a fractal agglomerate to the density of a Euclidean object of the same radius is thus given by the equation

3 $$ {\uprho}_{{\mathrm{a}}^{\hbox{'}}}/{\uprho}_{\mathrm{s}}=1/{\left({\mathrm{r}}_{\mathrm{a}}/{\mathrm{r}}_{\mathrm{particle}}\right)}^{0.5} $$

The apparent density of the agglomerate composed of COM spherical crystals with a diameter of 5.2 × 10^−6^ m (the density of COM is 2120 kg m^−3^ [http://www.chemicalbook.com/ChemicalProductProperty_EN_CB7154359.htm]) that can block the opening of the CD with a radius of 40 × 10^−6^ m was calculated to be ρ_a′_ = 2120 / (40 / 2.7)^0.5^ = 551 kg m^−3^. Such an agglomerate has void spaces filled with air. The apparent density of the agglomerate that contains a gas or liquid in void spaces among crystals is

4 $$ {\uprho}_{\mathrm{a}\hbox{'}}={\mathrm{F}}_{\mathrm{s}}{\uprho}_{\mathrm{s}}+{\mathrm{F}}_{\mathrm{g},1}{\uprho}_{\mathrm{g},1} $$

where F_g_,_l_ is the volume fraction of the agglomerate occupied by gas or liquid and F_s_ + F_g,l_ = 1. The volume fractions of COM agglomerate with radius of 40 × 10^−6^ m occupied by solid was calculated to be 0.260. The apparent density of the agglomerate with void space filled with liquid (i.e. when the agglomerate is submerged in urine) was then calculated to be 1302 kg m^−3^. The apparent density of COM agglomerates was previously estimated to be 1500 kg m^−3^ [17].

Any agglomerate with a radius smaller than 40 × 10^−6^ m (the radius of the opening of the CD) would be washed away by a stream of urine from the downward-draining Bellini duct (see below). The mass of a Euclidean object of this size and with the above-described density would be 5.68 × 10^−10^ kg and hence the mass of the solid fraction of the agglomerate would be 5.68 × 10^−10^ × 0.260 = 1.48 × 10^−10^ kg. Such an agglomerate would be composed of 948 crystals with a radius of 2.6 × 10^−6^ m. Assuming (the unrealistic case) that all of the crystals present in the Bellini’s duct create this agglomerate, the number concentration of the constituting crystals would be 948 / 5.94 × 10^−11^ = 1.6 × 10^13^ m^−3^ of urine.

The number concentration of COM crystals present in urine during crystalluria has been shown to be 2.41 × 10^10^ m^−3^ [17], and this value hence indicates the number concentration of nuclei formed at the beginning of the ascending loop of Henle, that is the maximum number concentration of COM crystals in urine. Since this concentration is about three orders of magnitude lower than the concentration necessary for forming blocking agglomerate, the CD of any orientation cannot be blocked by agglomerates of COM crystals.

The half-time τ_½_ of agglomeration, i.e. the time necessary for halving the number of separate particles present in a unit volume of suspension, N_o_, controlled by the laminar shear rate, is defined as [18]

5 $$ {\uptau}_{\frac{1}{2}}=1/\mathrm{A}\ {\mathrm{N}}_{\mathrm{o}} $$

with the A term given by

6 $$ \mathrm{A}=2\ \upalpha\ \mathrm{G}\ {\mathrm{r}}^3/3\ {\mathrm{k}}_{\mathrm{v}} $$

where r is the particle radius (m), α is the collision efficacy coefficient, k_v_ represents the volume shape factor and G is the shear rate (s^−1^) defined as

7 $$ \mathrm{G}=4\ \mathrm{Q}/{{\uppi \mathrm{r}}_{\mathrm{CD}}}^3 $$

where r_CD_ is a radius of the CD. For Q = 1.63 × 10^−11^ m^3^ s^−1^ (Table 1) and r_CD_ = 4 × 10^−5^ m, the shear rate would be 324 s^−1^. Taking α = 1 (i.e. each collision is successful), *r* = 2.6 × 10^−6^ m, k_v_ = 4 π/3 and N_o_ = 1.63 × 10^13^ m^−3^ then the half time would be 527 s. The kinetics of agglomeration showed that formation of the blocking agglomerate in a suspension containing enough particles to form the agglomerate would be substantially longer than the transit time through the CD.

Concentration of COM particles in the CD and kinetics of their agglomeration showed that any agglomerate of COM crystals formed in the CD cannot acquire a large enough size to be retained and block the Bellini’s duct.

### Effect of gravity

Due to the changing position of the body, all of the CDs are not necessarily always downward-draining, and some can be in an upward-draining position. In such a case an agglomerate of a sufficient size can be retained in the collecting duct due to settling.

A solid particle cannot be conveyed upward when the terminal settling velocity of the particle exceeds the fluid velocity. The terminal velocity is the highest velocity attainable by an object as it falls through a fluid. The terminal settling velocity, u_te_, of the particle in an unbounded fluid is given by the Stokes law if the Reynolds number of particle, Re_p_ = u_te_ D ρ_s_ / μ, was considerably smaller than unity

8 $$ {\mathrm{u}}_{\mathrm{te}}=\mathrm{g}\ \left({\uprho}_{\mathrm{s}}\hbox{--} {\uprho}_{\mathrm{l}}\right)\ {\mathrm{D}}^2/18\ \upmu $$

where g is the gravity acceleration 9.81 m s^−2^. Re_p_ of a spherical COM particle with a diameter 5.2 × 10^−6^ m would be 2.5 × 10^−3^ for u_te_ = 1.94 × 10^−5^ m s^−1^ estimated from eq.(8). Because the requisite condition for use of eq.(8), i.e. Re_p_ « 1, is satisfied, the calculated value of u_te_ represents the terminal settling velocity of the considered particle. Liquid in a distance 5.2 × 10^−6^ m from the edge of the CD opening is moving with a linear velocity 1.6 × 10^−3^ m s^−1^ (eq.1). Since this fluid velocity exceeds by about two orders of magnitude the terminal settling velocity of the largest COM crystal that can develop in the nephron, all individual COM crystals are invariably washed away from the collecting duct.

The size of a COM agglomerate with a density 1500 kg m^−3^ and having the same settling velocity as the average linear velocity of the liquid at the CD opening was estimated using eq.(8) to be 1 × 10^−4^ m and its Re_p_ was estimated to be 0.58. Because the condition Re_p_ « 1 is not fulfilled, the terminal settling velocity of such an agglomerate has to be determined by iterations from the set of expressions

9 $$ {\displaystyle \begin{array}{c}\hfill\ {\mathrm{Re}}_{\mathrm{p}}={\mathrm{u}}_{\mathrm{te}}\mathrm{D}\ {\uprho}_{\mathrm{s}}/\upmu \hfill \\ {}\hfill \mathrm{D}=3\ {{\mathrm{u}}_{\mathrm{te}}}^2{\mathrm{c}}_{\mathrm{D}}\ {\uprho}_{\mathrm{l}}/\left[4\ \left({\uprho}_{\mathrm{s}}\hbox{-} {\uprho}_{\mathrm{l}}\right)\ \mathrm{g}\right]\hfill \\ {}\hfill {\mathrm{c}}_{\mathrm{D}}=24/{\mathrm{Re}}_{\mathrm{p}}+4/\surd {\mathrm{Re}}_{\mathrm{p}}+0.4\hfill \end{array}} $$

where c_D_ is the drag coefficient [19]. The diameter of a spherical agglomerate of COM crystals with a density 1500 kg m^−3^ that can be retained in an upward-draining CD, i.e. having a settling velocity equal to or higher than the average linear velocity of the liquid at the CD opening, was calculated to be 8.86 × 10^−5^ m and Re_p_ was calculated to be 0.51. The size of this agglomerate exceeds the size of the CD opening. Therefore, any agglomerate of COM crystals formed in the CD has a sedimentation velocity lower than the velocity of liquid flow and would therefore be washed out into the calyx even from an upward-draining CD. Performed calculations thus indicate that CDs in any orientation cannot be blocked by any an agglomerate of COM crystals.

### Wall effect

The influence of walls on a settling particle, known as the wall effect, leads to an increase in the drag force exerted on a falling particle that results in a retarding effect on the terminal velocity of settling [20, 21]. The wall effect becomes important when the diameter of the particle becomes fairly noticeable with respect to the diameter of the tubule. In these cases the settling velocity calculated for an unbounded medium from eqs. (8,9) has to be corrected using the expression [22]

10 $$ {\mathrm{u}}_{\mathrm{te}}\left(\mathrm{corr}\right)={\mathrm{u}}_{\mathrm{te}}\left(\mathrm{eqs}.7,8\right)/\left[1\hbox{--} {\left(\mathrm{D}/2\mathrm{r}\right)}^{1.5}\right] $$

The settling velocity of larger particles is in fact lower than those previously calculated without considering the influence of the wall effect. This fact further diminishes the likelihood of a Randall’s plug forming by the free particle mechanism.

## Discussion

Formation of a Randall’s plug in the Bellini’s duct by the free particle mechanism assumes that a particle of sufficient size, be it a single crystal or agglomerate of smaller crystals, is retained in the duct and develops into the full-sized Randall’s plug. The feasibility of this mechanism was evaluated for a model kidney with a structure as described recently by Robertson. The composition of Randall’s plug consisting of COM crystals considered in the present analysis is identical with the composition considered in the analysis Robertson even though the plug can also contain or be composed of COD crystals [23]. The conditions prevailing in the nephron considered in the current analysis – the highest reported supersaturation with respect to COM, the lowest liquid flow, absence of inhibitors of COM crystallization, all crystals present in the CD having the same maximum achievable size and fast agglomeration of crystals into single particle – are all the most favourable for formation of Randall’s plug by the free particle mechanism.

Hydrodynamic conditions prevailing in the loop of Henle and distal tubule calculated above are in satisfactory agreement with the results of the Robertson model [3, 4]. The substantial discrepancy in the calculated transit time of liquid through the CD (4 s versus 50 s) is difficult to explain because the details of the Robertson model have not been published. However, the volumetric flow rate and the linear velocity of liquid at the opening of the CD derived from basic data - diuresis and number of CDs per kidney - are correct and hence were used in all calculations.

COM nuclei originating at the beginning of the ascending loop of Henle where the supersaturation with respect to COM attains value of 14 are expected to be formed by heterogeneous nucleation. This means that the number of formed primary particles is largely governed by the number of effective heteronuclei (organic debri) present in the liquid. A uniform spatial distribution of COM primary particles can be expected. Individual particles thus move with different velocities with a parabolic velocity profile according to their distance from the tubule wall. The velocity of particles increases from zero for particles close to the wall to double the average linear velocity of the liquid for particles in the centre of tubule. Particles close to the wall do not move, but grow because they are surrounded by moving supersaturated liquid of the adjoining shell. When the particle grows to the point at which it extends into the adjoining 2nd shell it starts moving with the velocity of this shell. This process repeats until the particle leaves the nephron and is washed out into the calyx. Maximum attainable size of COM individual particle at the opening of the CD travelling in the vicinity of tubule wall was calculated to be about five micrometers and its total transit time was calculated to be about 18 min assuming a constant flow of liquid. The settling velocity of particle was not considered in these calculations because it is negligible compared to the velocity of the liquid flow. The final size of the particle and its transit time through the nephron depend on the prevailing level of supersaturation. At higher supersaturation the final particle size is larger and the transit times shorter whereas at lower values it is vice versa. Even in specific cases, such as dramatically decreased production of urine when the transit time would be much longer, the size of individual COM particles would still be far smaller than the size necessary for blocking the CD. Experimental study of growth of CaOx crystals in urine-like solution led to similar conclusion [24].

The final size of COM particle depends on prevailing level of supersaturation and duration of its contact with supersaturated liquid, i.e. on its transit time through the nephron. Particle is carried by the liquid in assumed pure laminar flow. Pure laminar flow in the nephron is, to some extent, disturbed at sharp bends of tubule, in the vicinity of kinks present in the nephron and at termination of the nephron at the collecting duct. Impact of these irregularities influencing laminar flow on the transit time of particle is difficult to estimate because quantitative data describing these irregularities are not available. Moreover, it is doubtful that the chemical engineering treatment of these disturbances suitable for tubes of larger diameter is applicable for capillaries (nephron).

Isolated buoyant spheroidal particle in a uniform simple laminar shear flow at low Reynolds number does not move along the straight line but moves in closed orbits. The centre of the particle moves with the velocity of the undisturbed fluid at that point. Particle also tends to drift to the centre of a capillary [25]. Therefore a spheroidal primary particle initially situated in the close vicinity of tubular wall gradually drifts away from the wall and thus crosses over to the adjoining shell of faster moving liquid sooner than is expected in the used model based on the regular crystal growth. On the other hand, friction between particle and the tubular wall slows down the velocity of particle movement compared to the velocity of the respective liquid shell. Because no quantitative data are available, it was assumed that opposite effect of these two factors is roughly equal and negligibly influence calculated particle transit time through the nephron and hence the determined final particle size.

Flat prismatic COM particles predominantly appearing in urine tend to align with the laminar flow direction. But in regions where some turbulence appears, flat particles tend to adopt position perpendicular to the flow [26]. Particles aligned with the laminar flow move slower than model in Additional file 3 predicts, but particles perpendicular to the flow move faster than calculated. Based on the current knowledge it is impossible to estimate duration of each orientation during transit time of particles through the nephron. Effect of periodic changes in particle orientation on transit time was assumed to be balanced by assumption of spherical shape of particle used in calculations.

The growth rate of COM particles expressed by eq. (2) and used in the current analysis describes uninhibited growth that is when inhibitors of growth, such as citrate, magnesium, phytate, zinc, low-molecular weight peptides, certain amino acids etc. are absent. Since these inhibitory substances are actually always present in urine, though in individually different concentrations, the actual growth of COM particles in urine is generally lower than that given by eq.(2) and hence the real size of COM particles in the CD is smaller than those indicated by the above calculations.

Any single particle of COM formed in the nephron and CD at night, and during other situations when production of urine is low, cannot attain the size that can block the CD. An alternative mechanism in which a blocking object is formed by the agglomeration of COM particles in the CD was considered. Here, since the orientation of the kidney and hence of the CD frequently varies with the changing position of the human body two separate cases were examined: downward- and upward-draining positions of the CD.

CD in the downward-draining position can be blocked only if the blocking object was formed during the transit time of the liquid through the CD. An agglomerate with a radius 4 × 10^−5^ m can be theoretically retained at the beginning of CD and block it. Such an agglomerate cannot develop in the CD since it would consist of significantly more particles than the maximum possible number of particles present in the CD. Even if the required number of particles were present in the CD, the formation of an agglomerate of such size would be impossible within the given time frame from view-point of agglomeration kinetics. Smaller agglomerates originating in the CD cannot be retained in the Bellini’s duct and are without exception washed out into the calyx. A Randall’s plug thus cannot develop in downward-draining CDs by the free particle mechanism.

The settling velocity of particles must be considered in the upward-draining CDs because it diminishes the capability of a liquid stream to wash them out into the calyx. An individual COM particle cannot attain during transit through nephron diameter 6.7 × 10^−5^ m at which its settling velocity equals velocity of liquid stream at the opening of CD. An agglomerate would need to have a diameter of 8.86 × 10^−4^ m to display a terminal settling velocity equal to the linear velocity of a liquid at the CD opening, even without considering the retarding effect of the tubule wall on the settling velocity. Such a diameter does exceed the dimensions of the CD opening in the used kidney model. Moreover, number of crystals needed to form such a large agglomerate is about three orders of magnitude greater than maximum number present in the CD during the transit time. Therefore any agglomerate formed in the CD is smaller than the size of a blocking agglomerate and would be washed out of the CD regardless of its orientation. Transporting solid particles in vertical pipelines requires the bulk fluid velocity to be greater than twice the particle settling velocity [27] and this condition was fulfilled in our case.

Calculations showed that any COM particle that can be formed in the nephron, be it a single crystal or agglomerate, was too small to be retained unbounded in the CD of any orientation. The Randall’s plug thus cannot originate in the kidney with parameters corresponding to the used kidney model by the free particle mechanism even during period of decreased urine production such as at night.

All mammals display a peristaltic movement of renal calyces [28]. During a period of compression the CD experiences a contraction, which is periodically repeated, as a wave. As a result of this movement the papillae, and consequently the CD, are evidently subjected to some regular squeezing, to facilitate the expulsion of liquid and present solid objects. These features further support that a mechanism other than the free particle mechanism is responsible for the formation of the Randall’s plug.

The detail structure of the kidney has been observed to vary quite considerably from one individual to the next. The number of CDs per kidney has been observed to vary from 50 to 1120, and the diameter of the CD at its beginning and opening to vary from 2 × 10^−5^ to 3 × 10^−5^ m and from 8 × 10^−5^ to 2 × 10^−4^ m respectively [2]. The most favourable combination of kidney parameters for forming a Randall’s plug by the free particle mechanism would include 1120 CDs, a CD diameter of 2 × 10^−4^ m at its opening and a low urine production of 0.30 × 10^−3^ m^3^ per 8 h. This case corresponds to a volumetric flow rate of 4.65 × 10^−12^ m^3^ s^−1^ and a linear flow velocity of urine at the CD opening of 1.48 × 10^−4^ m s^−1^. The terminal settling velocity of a crystal with diameter 8 × 10^−6^ m located in the vicinity of wall would be equal to the linear flow velocity of fluid and would be retained in the upward-draining CD. Under favourable conditions - deficiency of inhibitors of crystal growth and presence of agglomeration promoters – such crystals by growth and fast agglomeration could form a blocking concretion before the position of the CD is changed from upward to downward pointing as a result of natural body movements.

The free particle mechanism may be responsible for the formation of a Randall’s plug in infrequent specific cases. However, the majority of incidences of renal papillary calcifications are caused by a different mechanism such as the fixed particle mechanism. A similar conclusion was reached by Finlayson and Reid [2] who analyzed the formation of the Randall’s plug using a probabilistic approach applied on a slightly different model of the kidney.

## Conclusions

Formation of a Randall’s plug composed of COM in the Bellini’s duct of kidney with structure described recently by Robertson by the free particle mechanism is rather improbable even under the most favourable conditions prevailing in the nephron – the highest reported supersaturation, the lowest typical urine production, absence of inhibitors of COM crystallization, maximum size of crystals present in the CD and fast agglomeration of crystals into one particle. The free particle mechanism may be responsible for the formation of a Randall’s plug composed of COM only in infrequent specific cases characterized namely by abnormal structure of kidney, dramatically decreased urine production, deficiency of inhibitors of crystal growth combined with the presence of agglomeration promoters and long-term upward-draining position of the CD. Majority of incidences of renal papillary calcifications are caused by other mechanisms such as the fixed particle mechanism.

## Additional files

Additional file 1: Used expressions. (DOCX 11 kb)

Additional file 2: Hydrodynamics of the CD. (DOCX 13 kb)

Additional file 3: The transit time of particle through the tubule. (DOCX 13 kb)

## Abbreviations

A: Area (m^2^)
c_D_: Drag coefficient
D: Diameter of particle (m)
D_m_: Mass agglomerate fractal dimension (−)
F: Volume fraction (−)
g: Gravity acceleration (m s^−2^)
G: Shear rate (s^−1^)
k_g_: Growth rate constant
k_v_: Volume shape factor (−)
L: Length (m)
N_o_: Number of separate particles present in a unit volume of suspension (m^−3^)
Q: Volumetric flow rate, i.e. volume of liquid passing tubule per second (m^3^ s^−1^)
R: Distance from the tubule axis (m)
r: Radius of tubule (m)
Re_p_: Reynolds number of particle (−)
Re: Reynolds number (−)
S: Supersaturation (−)
t: Time (s)
t_tr_: Transit time (s)
u: Linear velocity of liquid (m s^−1^)
u_te_: Terminal settling velocity of particle (m s^−1^)
V: Volume of tubule (m^3^)

## Greek symbols

τ: Time (s).
α: Collision efficacy coefficient (−).
ρ: Density (kg m^−3^).
μ: Dynamic viscosity (kg m^−1^ s^−1^).
τ_½_: Half time of agglomeration.
ρ_a′_: Apparent density of agglomerate - the ratio of mass of agglomerate to volume of Circumscribed sphere (kg m^−3^).

## Subscripts

a: Agglomerate
av: Average
g: Gas
l: Liquid

## Abbreviations

Ave: Average value of a function
CaOx: Calcium oxalate
CD: Collecting duct
COD: Calcium oxalate dihydrate
COM: Calcium oxalate monohydrate
