# Supplementary material for: Can Randall’s plug composed of calcium oxalate form via the free particle mechanism?
Source: BMC Urol. 2017 Sep 8;17:80. doi: 10.1186/s12894-017-0274-7 (PMC5591557; doi:10.1186/s12894-017-0274-7)
Supplement: Supplementary file 1 — Used expressions. (DOCX 11 kb) [file 12894_2017_274_MOESM1_ESM.docx]

Additional file 1 Used expressions

Volume of conical truncated cone V = (π/3) L (r_1_^2^ + r_1_r_2_ + r_2_^2^)

Average value of function f(x)

b

Ave[f(x)] = [1 / (b – a)] ∫ f(x) dx

a

Transition time t_tr_ = V/Q = L/u_av_

Re = 2 r u_av_ ρ / μ
