# Supplementary material for: Can Randall’s plug composed of calcium oxalate form via the free particle mechanism?
Source: BMC Urol. 2017 Sep 8;17:80. doi: 10.1186/s12894-017-0274-7 (PMC5591557; doi:10.1186/s12894-017-0274-7)
Supplement: Supplementary file 2 — Hydrodynamics of the CD. (DOCX 13 kb) [file 12894_2017_274_MOESM2_ESM.docx]

Additional file 2 Hydrodynamics of the CD

The surface of circular truncated cone of the length L is

A = π (r_1_ + r_2_) x [(L^2^ + (r_2_ – r_1_)^2^]^½^ (II.1)

The total area available for water absorption is the surface of three quarters of the CD length. From eq.(II.1) with r_1_ = 1.75x10^-5^ m, r_2_ = 4x10^-5^ m and L = 2.025x10^-2^ m, this surface is

A(tot) = 3.656x10^-6^ m^2^

The surface available for water absorption in a distance L from the beginning of the CD is

A(L) = π (2.75x10^-5^ + 1.111x10^-3^ L)(L – 6.75x10^-3^) m^2^ (II.2)

90% of the liquid entering the CD at night, i.e. 0.9 x 1.63x10^-10^ = 1.467x10^-10^ m^3^ s^-1^, must be reabsorbed through A(tot), this is 1.467x10^-10^ / 3.656x10^-6^ = 4.01x10^-5^ m^3^ m^-2^ s^-1^ of water must be reabsorbed through unit surface of the CD. The volumetric flow rate in a distance L from the beginning of the CD is

Q(L) = 1.63x10^-10^ – A(L) x 4.01x10^-5^ m^3^ s^-1^ (II.3)

Combination of eqs. (II.2) and (II.3) gives the volumetric flow rate Q(L) in a distance L from the beginning of the CD

Q(L) = 1.86x10^-10^ – 1.40x10^-7^ L^2^ – 2.52x10^-9^ L m^3^ s^-1^  (II.4)

The average volumetric flow rate in the CD is

0.027

Ave[Q(L)] = [1 / (0.027 – 0.00675)] **∫**Q(L) dL = 7.58x10^-11^ m^3^ s^-1^

0.00675

The transit time of liquid through the CD is V(CD) / Ave[Q(L)] = 0.8 s.

The average linear flow rate in the CD is

u_av_ = (r_2_^3^ – r_1_^3^) /{3 (r_2_ – r_1_) x Ave[Q(L)]} = 3.45x10^-2^ m s^-1^
