# Supplementary material for: Can Randall’s plug composed of calcium oxalate form via the free particle mechanism?
Source: BMC Urol. 2017 Sep 8;17:80. doi: 10.1186/s12894-017-0274-7 (PMC5591557; doi:10.1186/s12894-017-0274-7)
Supplement: Supplementary file 3 — The transit time of particle through the tubule. (DOCX 13 kb) [file 12894_2017_274_MOESM3_ESM.docx]

**Additional file 3.** The transit time of particle through the tubule

The distance travelled by a particle in the n^th^ shell is

λ(n) = 2 u_av_ τ [1 – (R/r)^2^] (III.1)

where

τ = c / k_g_ (S – 1)^2^ (III.2)

τ is time before particle reaches the next shell due to its growth, and

R = r – (n – 1) c (III.3)

c is the n^th^ shell thickness 1x10^-7^ m. The total distance, L, travelled by a particle is

L = Σ λ(n) (III.4)

k

n=1

where k is the number of shells that particle growing from the initial size to the final size enters. Substituting eqs.(III.1,2,3) into eq.(III.4) and using Σ n = k (k + 1) / 2 and Σ n^2^ = k (k + 1) (2k + 1) / 6 gives

n=1

n=1

k

k

L / Z = – A k^3^ + B k^2^ – C k (III.5)

with coefficients

A = c^2^ / 3 r^2^

B = [2 c r + c^2^)] / 2 r^2^

C = (6 c r + c^2^) / 6 r^2^

Z = 2 u_av_ c / [k_g_ (S – 1)^2^]

Solving eq.(III.5) for L equal to the length of respective tubule gives number of shells that particle reaches during travelling in the tubule. The transit time is then t_tr_ = k x τ.
